# Editorial: Sulfate Radical-Based Advanced Oxidation Processes for Water and Wastewater Treatment

**DOI:** 10.3389/fchem.2021.691005

**Published:** 2021-04-30

**Authors:** Wei Liangliang, Zhang Guangshan, Zhang Huichun

**Affiliations:** ^1^State Key Laboratory of Urban Water Resources and Environment (SKLUWRE), School of Environment, Harbin Institute of Technology, Harbin, China; ^2^College of Resource and Environment, Qingdao Agricultural University, Qingdao, China; ^3^Department of Civil and Environmental Engineering, Case Western Reserve University, Cleveland, OH, United States

**Keywords:** sulfate radicals, wastewater treatment, activation methods, degradation mechanism, intermediates and kinetics

In many industrial wastewater/natural water treatment processes, traditional biological treatment techniques do not work well owing to the high toxicity, non-biodegradable and refractory chemical characteristics of those wastewaters, as well as the abundant existence of organic substances. Sulfate radical-based advanced oxidation processes (SR-AOPs) have been proven to be an alternative approach to eliminating refractory organic contaminants because sulfate radicals have the advantages of high redox potential, long half-life and wide pH applicability. However, the oxidants persulfate (PS) and peroxymonosulfate (PMS) cannot undergo self-decomposition to generate sulfate radicals by themselves, because of their low reaction rates. Hence, developing effective and economical activation methods to efficiently generate sulfate radicals for water and wastewater treatment and refractory organics removal has been a hot topic in recent years.

In this perspective, the special issue of “Sulfate Radical-Based Advanced Oxidation Processes for Water and Wastewater Treatment” encompasses a collection of original research and review articles focusing on refractory pollutant degradation. The focal points are to report the activation methods for PS/PMS, their working mechanisms, kinetics of sulfate radicals on organics elimination, and potential intermediates during the degradation process. This collection of articles features one review and four research papers.

The review summarizes typical activation methods, including physical methods (such as heating, UV, and ultrasound), chemical approaches (including transition metal ions, alkaline conditions), and coupled activation methods, for efficient sulfate radicals generation. Moreover, their applications and economic feasibility are evaluated (Xia et al.). The degradation performance of PS/PMS oxidation for typical domestic and industrial wastewater treatment, especially for refractory organics degradation, has been summarized. The categories and characteristics of the intermediates are also evaluated. Based on the above, roles of sulfate radicals, their kinetic characteristics, and possible mechanisms for organic elimination are discussed.

Quinones and organic matter, which contains quinone-like groups, have been found to be effective as an accelerator or activator in advanced oxidation processes. To obtain the most suitable target chemicals and the optimal working conditions of quinone-activated persulfate systems, one of the original research papers in this collection (Shi et al.) investigates how the reaction conditions of pH, quinone species, and concentration affected the quinone-activated persulfate oxidation. The optimal operational parameters were also obtained. In addition, a quantitative structure-activity relationship model was established based on the evaluation of the degradation of 15 monoaromatics, and the experimental results revealed that the more negative atomic net charges on carbon atoms are, the lower the degradation rate. In short, chemicals with a smaller ${\text{q}}_{\text{C}}^{-}$ (the largest negative partial net charge on a carbon atom) are more easily oxidized in the quinone-activated persulfate system.

The work by Wang X. et al. evaluates the performance of a novel heterogeneous oxidation system, biochar supported nano-zero valent iron (BC/nZVI), for PS activation and nitrochlorobenzene (NCB) degradation. Briefly, the degradation kinetics of m-, p-, and o-NCB isomers in the aqueous phase were investigated. The effects of BC/nZVI composition, mole ratio of PS/(BC/nZVI), and the initial concentration of NCB on the performance of the oxidation were also examined. It was found that the optimal operation condition was under a Fe/BC/PS mole ratio of 1:1:1 and the initial NCB concentration of 10 mg/L.

To simultaneously remove algae and micro-organic pollutants in natural water, UV/PS was firstly applied to simultaneously eliminate Microcystis aeruginosa (*M. aeruginosa*) and 2,4,6-trichlorophenol (TCP) via bench scale tests (Wang J. et al.). Experimental results revealed that both *M. aeruginosa* and TCP could be efficiently removed during the UV/PS process. Specifically, the oxidation of ${\text{SO}}_{4}^{-}\cdot$ and the direct photolysis of UV played a key role in TCP degradation, whereas OH· and UV direct photolysis were responsible for the *M. aeruginosa* removal. In addition, the removal performance of the released intracellular organics from algae cells was inhibited in the presence of TCP. However, the inhibitory effect could be neglected under a high PS dose (>1.5 mM).

The decontamination kinetics of various target pollutants (benzoic acid, nitrobenzene, and trichloromethane) and the oxidation by-products (bromate and chlorate) of three UV/peroxide processes (UV/PMS, UV/PS, UV/H_2_O_2_) are compared in the study of Guan et al. They found that those three oxidation systems exhibited a quite different removal trend for different target pollutants under varied pH conditions (acidic, neutral, or alkaline). For benzoic acid and nitrobenzene degradation, UV/H_2_O_2_ was the most efficient one among the three systems under both acid and natural conditions, while UV/PMS showed the best removal performance under alkaline conditions. For trichloromethane removal, UV/PS was the most efficient under all pH conditions. The amounts of bromate and chlorate formed in the above three systems varied widely in different aqueous environments. Furthermore, the inhibition degree was compared for the three UV/peroxide processes in the presence of common radical scavengers, including bicarbonate, carbonate, and natural organic matter (NOM).

As guest editors, we would like to thank all the authors/contributors for their valuable contributions to this Research Topic and all the reviewers for their important and thoughtful comments and insights. We believe that the present article collection illustrates some of recent advances in SR-AOPs for water and wastewater treatment. At the same time, we hope that this Research Topic will provide useful insight into the PS/PMS activators development and refractory organics degradation.

## Author Contributions

All authors listed have made a substantial, direct and intellectual contribution to the work, and approved it for publication.

## Conflict of Interest

The authors declare that the research was conducted in the absence of any commercial or financial relationships that could be construed as a potential conflict of interest.

